# Supplementary material for: A predictive approach to integrating connectivity into landscape scale protected areas planning
Source: PLoS One. 2026 Apr 29;21(4):e0346336. doi: 10.1371/journal.pone.0346336 (PMC13127925; doi:10.1371/journal.pone.0346336)
Supplement: S1 Table — See Pither et al. [31] for a detailed description of landscape feature classifications and data layers used. (DOCX) [file pone.0346336.s001.docx]

| **cost value** | **landscape features** |
| --- | --- |
| 0.1 | natural areas^*^ within protected areas boundaries |
| 1 | natural areas^*^ outside of protected areas boundaries |
| 10 | minor roads, pasturelands, forestry (cuts < 35 yrs old) |
| 100 | croplands, two-lane highways |
| 1000 | cities, railroads, multi-lane highways lakes (≧10 ha), rivers (flow > 28 m^3^/sec), mines, dams, nighttime lights |

*natural areas refers to all terrestrial, non-anthropogenic landscape features including forests, wetlands, grasslands, etc.
