# Supplementary material for: A predictive approach to integrating connectivity into landscape scale protected areas planning
Source: PLoS One. 2026 Apr 29;21(4):e0346336. doi: 10.1371/journal.pone.0346336 (PMC13127925; doi:10.1371/journal.pone.0346336)
Supplement: S2 Table — Buffer size indicates the size of buffer placed around generic parks when calculating landscape context variables. A range of buffer sizes were tested to determine the scale of effect of landscape context variables. Model variable codes represent: EAR = edge-to-area ratio; Dist_Cent = distance to center; Dist_Park = distance to nearest park; Dist_Node = distance to nearest sentinel node; PropAnth_Park = proportion of anthropogenic development within parks; PropWater_Park = proportion of water within parks; PropAnth_# = proportion of anthropogenic development within a buffer surrounding parks (one of 2, 10, 25, or 50 km); and PropWater_# = proportion of water within a buffer surrounding parks (one of 2, 10, 25, or 50 km). (DOCX) [file pone.0346336.s002.docx]

| **Model #** | **Model Type** | **Buffer Size (km)** | **Predictors** |
| --- | --- | --- | --- |
| 1 | Null | - | Intercept only |
| 2 | Distance | - | EAR + Dist_Cent + Dist_Park + Dist_Node |
| 3 | Landscape Context | 2 | EAR + PropAnth_2 + PropWater_2 + PropAnth_Park + PropWater_Park |
| 4 |  | 10 | EAR + PropAnth_10 + PropWater_10 + PropAnth_Park + PropWater_Park |
| 5 |  | 25 | EAR + PropAnth_25 + PropWater_25 + PropAnth_Park + PropWater_Park |
| 6 |  | 50 | EAR + PropAnth_50 + PropWater_50 + PropAnth_Park + PropWater_Park |
| 7 | Global | 2 | EAR + Dist_Cent + Dist_Park + Dist_Node + PropAnth_2 + PropWater_2 + PropAnth_Park + PropWater_Park |
| 8 |  | 10 | EAR + Dist_Cent + Dist_Park + Dist_Node + PropAnth_10 + PropWater_10 + PropAnth_Park + PropWater_Park |
| 9 |  | 25 | EAR + Dist_Cent + Dist_Park + Dist_Node + PropAnth_25 + PropWater_25 + PropAnth_Park + PropWater_Park |
| 10 |  | 50 | EAR + Dist_Cent + Dist_Park + Dist_Node + PropAnth_50 + PropWater_50 + PropAnth_Park + PropWater_Park |
