# Supplementary material for: A predictive approach to integrating connectivity into landscape scale protected areas planning
Source: PLoS One. 2026 Apr 29;21(4):e0346336. doi: 10.1371/journal.pone.0346336 (PMC13127925; doi:10.1371/journal.pone.0346336)
Supplement: S3 Table — Model number corresponds to those used in S2 Table (DOCX) [file pone.0346336.s003.docx]

| **Model #** | **Model Type** | **K** | **AIC_c_** | **∆AIC_c_** | **AIC_c_ weight** | **Log likelihood** |
| --- | --- | --- | --- | --- | --- | --- |
| 7 | Global | 10 | 160.58 | 0.00 | 0.85 | -67.47 |
| 8 | Global | 10 | 165.48 | 4.91 | 0.07 | -69.92 |
| 10 | Global | 10 | 166.39 | 5.82 | 0.05 | -70.38 |
| 9 | Global | 10 | 167.75 | 7.18 | 0.02 | -71.05 |
| 2 | Distance | 6 | 174.66 | 14.08 | 0.00 | -80.35 |
| 6 | Landscape Context | 7 | 176.16 | 15.58 | 0.00 | -79.75 |
| 4 | Landscape Context | 7 | 179.66 | 19.08 | 0.00 | -81.50 |
| 5 | Landscape Context | 7 | 179.70 | 19.12 | 0.00 | -81.52 |
| 3 | Landscape Context | 7 | 180.29 | 19.72 | 0.00 | -81.81 |
| 1 | Null | 2 | 188.87 | 28.29 | 0.00 | -92.31 |
