# Supplementary material for: A predictive approach to integrating connectivity into landscape scale protected areas planning
Source: PLoS One. 2026 Apr 29;21(4):e0346336. doi: 10.1371/journal.pone.0346336 (PMC13127925; doi:10.1371/journal.pone.0346336)
Supplement: Attachment — Submitted filename: Responses_to_Reviewers.docx [file pone.0346336.s005.docx]

Journal Requirements:

When submitting your revision, we need you to address these additional requirements.

1.Please ensure that your manuscript meets PLOS ONE's style requirements, including those for file naming. The PLOS ONE style templates can be found at

<https://journals.plos.org/plosone/s/file?id=wjVg/PLOSOne_formatting_sample_main_body.pdf> and

[https://journals.plos.org/plosone/s/file?id=ba62/PLOSOne_formatting_sample_title_authors_affiliations.pdf](https://track.editorialmanager.com/CL0/https:%2F%2Fjournals.plos.org%2Fplosone%2Fs%2Ffile%3Fid=ba62%2FPLOSOne_formatting_sample_title_authors_affiliations.pdf/1/010f0198d090a782-1acb11d3-568b-4904-847f-295b2f665b31-000000/DXs-APkjNmj_eLQ_yy8FICovfjwu75c4kC3hRHji3Og=225)

**Response 1:** We have revised the manuscript to meet style requirements.

2. Thank you for stating the following financial disclosure:

“Funding for this research was provided by the Ontario Ministry of the Environment, Conservation, and Parks and the Ontario Ministry of Natural Resources.”

Please state what role the funders took in the study.  If the funders had no role, please state: "The funders had no role in study design, data collection and analysis, decision to publish, or preparation of the manuscript."

If this statement is not correct you must amend it as needed.

Please include this amended Role of Funder statement in your cover letter; we will change the online submission form on your behalf.

**Response 2:** We have included the adjusted financial statement in our cover letter as suggested.

3. When completing the data availability statement of the submission form, you indicated that you will make your data available on acceptance. We strongly recommend all authors decide on a data sharing plan before acceptance, as the process can be lengthy and hold up publication timelines. Please note that, though access restrictions are acceptable now, your entire data will need to be made freely accessible if your manuscript is accepted for publication. This policy applies to all data except where public deposition would breach compliance with the protocol approved by your research ethics board. If you are unable to adhere to our open data policy, please kindly revise your statement to explain your reasoning and we will seek the editor's input on an exemption. Please be assured that, once you have provided your new statement, the assessment of your exemption will not hold up the peer review process.

**Response 3:** Our data is stored on an online repository as indicated in our data availability statement and will be publicly available upon acceptance of the manuscript.

4. We note that Figure 1, 2 and 3 in your submission contain map images which may be copyrighted. All PLOS content is published under the Creative Commons Attribution License (CC BY 4.0), which means that the manuscript, images, and Supporting Information files will be freely available online, and any third party is permitted to access, download, copy, distribute, and use these materials in any way, even commercially, with proper attribution. For these reasons, we cannot publish previously copyrighted maps or satellite images created using proprietary data, such as Google software (Google Maps, Street View, and Earth). For more information, see our copyright guidelines: <http://journals.plos.org/plosone/s/licenses-and-copyright>.

**Response 4:** We have included the necessary attribution statements for these figures within the figure captions and directly within the map for Figure 2.

5. Please include captions for your Supporting Information files at the end of your manuscript, and update any in-text citations to match accordingly. Please see our Supporting Information guidelines for more information: <http://journals.plos.org/plosone/s/supporting-information>.

**Response 5:** We have moved the Supporting Information section to the end as suggested.

6. If the reviewer comments include a recommendation to cite specific previously published works, please review and evaluate these publications to determine whether they are relevant and should be cited. There is no requirement to cite these works unless the editor has indicated otherwise.

**Response 6:** No citations were recommended.

7. Please review your reference list to ensure that it is complete and correct. If you have cited papers that have been retracted, please include the rationale for doing so in the manuscript text, or remove these references and replace them with relevant current references. Any changes to the reference list should be mentioned in the rebuttal letter that accompanies your revised manuscript. If you need to cite a retracted article, indicate the article’s retracted status in the References list and also include a citation and full reference for the retraction notice.

**Response 7:** We have reviewed our reference list, and everything is complete and correct.

Additional Editor Comments:

Dear Dr. O'Brien,

After this review round, both reviewers believe your manuscript is almost ready for acceptance. Therefore, please apply the suggestions made by the reviewers and resubmit your manuscript and I am sure the text will be accepted for publication in PLoS One.

Sincerely,
Daniel Silva

**Reviewer #1:**

I enjoyed reading the manuscript by O'Brien and collaborators. They studied an alternative method to predict protected areas (PAs) network connectivity, measured connectivity using simulated PAs, and provided PA characteristics that benefit connectivity. The density values maps were an excellent output of the tool presented and are very suitable for application in decision-making on conservation planning. The manuscript is well-written and has a robust general design.

**Response 8:** We are glad to hear the reviewer enjoyed our manuscript and appreciate the provided suggestions. We have done our best to incorporate all suggestions, which we think has greatly improved our manuscript.

After addressing a few issues, I found this article suitable for publication in Plos One.
One of my concerns is the lack of a practical perspective on how this method (and tool) will be made available to stakeholders who are central to protected area planning. While I understand that this was not the primary goal of the study, providing insights into how the tool will be delivered to key stakeholders could help bridge the gap between academic ideas and practical implementation. Without this, the method risks being another valuable yet underutilized concept published in the literature.
Additionally, some information requires a detailed explanation instead of being limited to a single sentence or citation. For instance, "well-connected" and Canadian protected area networks. The first terminology appears multiple times in the text, however, what is its meaning? It would be helpful to specify the criteria or requirements for connectivity in this context. For the second, it would be beneficial for international readers to include a brief explanation of how PA establishment works in Canada, as this process varies significantly between countries. Further, the five cost ranks were not well explained. 0.1 is the value within the PA and 1000 is the value on a very anthropogenic landscape, but the intermediate values remain unclear. Finally, a schematic figure summarizing the methods could be a valuable addition, helping readers better understand the sequence of analyses performed.

**Response 9:** Thank you for bringing these issues to our attention. We have done our best to address these below in the reviewer’s specific comments.

In the next few lines, I detail smaller issues:

Abstract - concise and well-written. The only lack is to provide a practical view of how this tool will be available to the decision-making actors.

**Response 10:** Thank you. We agree that a practical view of how this tool will be made available is important. We consider that we do not have space to add this to the abstract, but we have added some information on this in the discussion (L428-431).

L.83 - "(in fact, likely the opposite)" - Require an explanation.

**Response 11:** We have added some explanation here (L82-83).

L.106 / 119- The terminology "well-connected" needs to be explained.

**Response 12:** We have provided a definition at L88-91.

L.124 -Please, provide the reader with the difference between generic parks and PA networks. Were generic parks the simulated ones? The sentence is not clear.

**Response 13:** We have added some clarification here (L126).

L. 128 - Why did you prefer to use random PAs within the study site than to choose characteristics that are important (size, edge, distance to other PA, etc) to PA effectiveness?

**Response 14:** As outlined in previous research (O’Brien et al. 2023), we preferred a random selection procedure of sentinel node parks to ensure a relatively even distribution of nodes across the landscape rather than selecting parks based on other criteria (e.g., size), which may lead to a bias in node location and clustering of nodes. We have added a sentence to clarify this point (L132-135).

L. 128-133- The PAs establishment is different around the world. For comparison reasons, it is essential to provide details on how this works in Canada. In addition, in L.212-213, the broad readership does not know about it.

**Response 15:** Thank you for the suggestion. We think that it is not essential here to understand PA establishment in Canada, given that many PAs can be national, provincial/territorial, and private, and so there is not a single “Canadian” process for PA establishment. However, we have now provided a short description of the Canadian Protected Areas Database (CPCAD) to help clarify to the broad readership what this includes (L140-142), to help understand the Canadian context.

As for L212-213, we consider the exact details of these sites is not critical, but most important as we outline in the following lines (L235-237) that they represent “natural areas that are not currently protected but have been identified using characteristics making them suitable for protection”. In addition, we have now provided a link which provides more detail for the Areas of Natural and Scientific Interest (ANSIs; L234). Hopefully this provides more clarity to those readers who may be interested.

L. 137 - Provide information on the 5 cost ranks. Include it in the legend of Figure 1.

**Response 16:** We have now included a table in the supplemental material (S1 Table) that provides more details on the 5 cost ranks.

L. 159 - Why did you choose to work with small-sized (6.25 km²) PAs?

**Response 17:** As described in L176-177, we selected this size of park to match the average size of PAs in this region, which tend to be small.

L. 180 - Which ones?

**Response 18:** Assuming the reviewer is asking which generic parks were added, we added each to the landscape independently of one another to determine how the addition of each influenced connectivity of the PA network. Or perhaps the reviewer is wondering if the generic parks are the simulated or real parks? If so, we have added text to clarify that here we are referring to the simulated parks (L197).

L. 248-249 - Interesting result. A discussion on this is needed.

**Response 19:** Indeed, we agree this warrants some discussion. We have added some lines interpreting these results to the discussion (L383-387).

L. 295-298 - Repeated information, is not necessary.

**Response 20:** We have removed this as suggested.

L. 306-308 - I agree. However, to make the bridge between academics and conservation in practice, how to deliver this approach in a practical way?

**Response 21:** This is a great point. We have added some discussion of this at L428-431. We also note that we have already shared this tool directly with stakeholders through a series of workshops, which we highlight now (L429-431). The code and data required to run the predictive model described in this manuscript is also available online at <https://figshare.com/s/17e67a7fafa68b5af9ec>, which we provide the ‘Data Availability’ section.

L. 317-327 - To this discussion, movement data (Movebank database) on known species could be simulated, and this may be the next step of this study.

**Response 22:** Indeed, the effectiveness of different sized protected areas in terms of connectivity is likely to vary with species. We have added some text to discuss how this species-specific relationship could be explored in the future (L353-354).

L. 332-337 - Is there any more bias linked to the nodes? This info may fit better in the Method Section.

**Response 23:** Given this information is discussing results of the modelling, we consider that it makes sense to keep this info in the discussion rather than move to the methods.

L. 393- 410 - Very nice paragraph.

**Response 24:** Thank you.

Figure 2 needs quality improvements.

**Response 25:** We have done this.

Table S1 fits better in the main text, very important information.

**Response 26:** We have moved this table to the main text as suggested. This is now Table 1 in the revised manuscript

**Reviewer #2:**

General Evaluation
The manuscript reports original research on integrating connectivity measures into conservation planning. The study presents relevant results that may inspire replication in other contexts. The proposed strategy for addressing functional connectivity can be objectively implemented into conservation/restoration targets, and thus constitutes a valuable contribution to conservation science.
The manuscript has significant merit and should be considered for publication in PLOS One after the authors address the methodological clarifications, improve figure quality, and strengthen the interpretation of results. Below I provide some specific recommendations:

**Response 26:** Thank you for your kind words. We have done our best to address your recommendations, which we think has greatly improved our manuscript.

Materials and Methods
Line 129: Provide a clear rationale for the selection of 20 sentinel nodes. Explain how this choice could be adapted in landscapes with different numbers of nodes or spatial extents (e.g., 2,500 nodes or 50 nodes). Clarify whether micro-fragments are included.

**Response 27:**  We followed Koen et al. (2014), who showed that 15-20 nodes was sufficient for modelling omnidirectional connectivity across a similar region. We have added some explanation of this at L135-138. We suspect that larger landscapes (i.e., with more pixels) may require more nodes, but this is a research question that remains to be answered. Regarding the inclusion of micro-fragments, we considered a protected area for selection as a sentinel node so long as it was larger than or equal to the size of a pixel (i.e., 100m^2^). We have added a line to clarify this (L131-132).

Line 137: Explicitly classify all cost values. Which correspond to natural areas (e.g., 0.1), and what about the others?

**Response 28:**  Please see Response 16 to Reviewer 1.

Lines 156–157: Specify whether the 50 new generic parks represent intact natural areas or partially degraded areas. Detail the criteria for distance within the randomization process.

**Response 29:** The 50 generic parks contained a mix of natural and developed areas. The only criteria was that the centroid pixel be classified as natural. We have added some text to clarify this (L171-173). The randomization algorithm used a 50km distance value to ensure selected pixels were at least 50km apart. This was to ensure that there was no overlap between generic parks when calculating landscape variables surrounding parks. We have clarified this at L168-175.

Line 156: Remove the term “randomly”, since the parks were selected according to a 50 km distance criterion and are evenly distributed. Justify why 50 parks were chosen.

**Response 30:** We have kept the term “randomly” because parks were still selected according to a stratified random procedure from available spatial points. Specifically, our algorithm randomly selected 50 spatial points where a stratum was applied to the selection procedure, ensuring that points were at least 50 km apart. We have added text to clarify this in the manuscript (L169-171). We have also removed the term “evenly”, which has given the impression that generic parks were spaced exactly 50km apart from each other when they were actually at least 50km apart, but potentially more.

50 generic parks were selected because we considered that would provide a sufficient sample size for our models and would not be too computationally demanding. We have added some text on this justification (L166-167).

Lines 163–165: Specify the basis for the variety of possible additions of protected areas (e.g., vegetation type).

**Response 31:**  We have added some text here to clarify that what we meant here was that the selection of generic parks provided a range of values for the calculated model variables (L180).

Lines 165–168: Revise this unclear sentence. If generic parks reduce costs from 1 to 0.1, but natural areas were already 0.1, clarification is needed.

**Response 32:** Indeed, we understand the confusion. Additional clarification on the assignment of cost values as described in Response 16 and Response 28, should clear up confusion here.

Figures and Tables
Figure 1: Improve resolution and add labels directly in the image. Present one map with natural areas, sentinel nodes, and generic parks overlaid, and another with cost analysis results.

**Response 33:** We have improved the image quality and added labels to the image directly but have decided to present the map in a single image as previously submitted.

Figure 2: Replace with a higher-resolution map.

**Response 34:** Done.

Figure 3: Make the map more self-explanatory by replacing “A”/“B” with “top candidate parks” / “bottom candidate parks” and labeling black dots as “candidate parks”.

**Response 35:** An excellent suggestion, thank you.

Table 1: Report that the edge-area-ratio yielded p = 0.09 under a 95% threshold (non-significant), but would be significant under a 90% threshold. This should be mentioned and its implications discussed.

**Response 36:** We have added text at L334-335 to address this point.

Discussion
Lines 303–304: Avoid using sentinel node proximity as a conservation parameter, as sentinel nodes are analytical constructs.

**Response 37:** Indeed. We noted that the inclusion of this parameter in our model was to account for potential biases and suggest practitioners consider this in their interpretation of results (L366-368)

Lines 317–318: Specify which side of the SLOSS debate the results support (single large vs. several small).

**Response 38:** We have added some text here (Line 343-344).

Lines 317–327: Specify which connectivity model is most suitable for the study region.

**Response 39:** As stated in paper (L339-351), a single large paper would improve connectivity the most over several small protected areas, but one needs to also consider SLASS that shows one large single protected area as well as several small, protected areas.

Lines 354–356: Specify that, while natural areas adjacent to developed areas may contribute more to increasing overall connectivity, intact areas remain essential under other conservation criteria (e.g., species restricted to intact habitats).

**Response 40:**  Thank you. We have made sure to clarify this point (L382-383).

Lines 395–397: Revise the apparent contradiction regarding temporal applicability. Specify the temporal scale at which the method can project future scenarios (medium- or long-term), and what adjustments would be needed to account for future conditions.

**Response 41:** We have added some lines to indicate a suggested timeline for re-running the model to account for future conditions (L436-439).

Conclusion
Emphasize how this approach differs from existing methods (e.g., Integral Index of Connectivity in Conefor, or direct resistance values).

**Response 42:** We have added some lines to emphasize the differences (L415-419).

Highlight the novelty of prioritizing areas based on their potential to increase overall connectivity when added to the conservation network.

**Response 43:** We now highlight this in the discussion. See Response 42 above.
