# Supplementary material for: A predictive approach to integrating connectivity into landscape scale protected areas planning
Source: PLoS One. 2026 Apr 29;21(4):e0346336. doi: 10.1371/journal.pone.0346336 (PMC13127925; doi:10.1371/journal.pone.0346336)
Supplement: Attachment — Submitted filename: ResponseToReviewers.docx [file pone.0346336.s006.docx]

Dear Dr. O'Brien,

After this new review round, the reviewer believes your work is nearly suitable for publication in PLoS One after minor reviews are implemented. I hope the provided suggestions help you to improve your text. Once you finish implementing them, the work will be ready to be sent out for production.

Sincerely,

Daniel Silva

**Response 1:** We thank the editor for the opportunity to revise our manuscript. We think the provided suggestions have greatly improved the manuscript. We look forward to continuing the process towards publication at PloS One.

Reviewers' comments:

Reviewer #2:

The manuscript PONE-D-24-48938R1 presents important results for implementing connectivity in conservation planning, with strong policy relevance and timely alignment with the Kunming–Montreal GBF and 30 × 30 targets. It also presents a clear methodological framework with strong practical applicability for managers and practitioners. I recommend acceptance of the manuscript in PLOS ONE, following minor revisions to address some writing details. Below I provide several comments that can be addressed with additional clarification in the text:

**Response 2:** We are delighted to hear that the reviewer sees the importance and practical applicability of our method, and we thank them for their insightful comments on our manuscript. We have done our best to address these revisions below, which we consider has improved our manuscript.

The manuscript presents an approach that is theoretically well established in the connectivity and conservation planning literature. However, it provides a meaningful methodological increment by integrating techniques such as the use of sentinel nodes, MPER, simulations, and regression to predict connectivity gains from new protected areas. This methodological extension is particularly valuable for practical applications, as it creates a simplified model that managers can use to prioritize candidate areas based on site and landscape attributes. I believe that the methodological novelty is not sufficiently clear in the Introduction, which may confuse readers, given that the underlying techniques and metrics are well known.

To better specify the innovation of the study, I suggest replacing the phrases “we developed a novel predictive model” (lines 454–455) and “we highlight the novelty of our approach” (line 411) with “we extend the sentinel node framework by developing a predictive surrogate model for prioritization” and “we provide an operational tool to translate connectivity metrics into planning-relevant rankings”, respectively.

**Response 3:** Thank you for the suggestion. We have added this new text (L412-414 & L467-468).

The cost–benefit relationship of this approach relative to other techniques (e.g., Marxan and Zonation) is not clearly discussed and should be clarified in the Discussion.

**Response 4:** We have added some text at lines 406-441.

The predictive model is based on a relatively small training dataset (n = 50, with 8 predictors), which may limit its generalizability due to potential overfitting. I suggest acknowledging this more explicitly in the Methods.

**Response 5:** We have added more text to the Methods as suggested (L227-232)

The title would benefit from emphasizing the practical applicability of the study, for example: “A predictive approach for incorporating connectivity into protected areas planning”.

**Response 6:** We have modified the title as suggested.
